# Supplementary material for: DNA-bridging by an archaeal histone variant via a unique tetramerisation interface
Source: Commun Biol. 2023 Sep 22;6:968. doi: 10.1038/s42003-023-05348-2 (PMC10516927; doi:10.1038/s42003-023-05348-2)
Supplement: Supplementary file 2 — Supplementary Information [file 42003_2023_5348_MOESM2_ESM.pdf]

Supplementary Information

DNA-bridging by an archaeal histone variant via a unique tetramerisation interface

Sapir Ofer, Fabian Blombach, Amanda M. Erkelens, Declan Barker, Zoja Soloviev, Samuel Schwab, Katherine Smollett, Dorota Matelska, Thomas Fouqueau, Nico van der Vis, Nicholas A. Kent, Konstantinos Thalassinos, Remus T. Dame, and Finn Werner

Supplementary Table 1

List of oligonucleotides used in this study

Oligo ID	Oligo name	SEQUENCE	Purpose (resulting plasmid)
Heterologous expression of MJ1647, A3 and variants			
**FW697**	MJ1647 NdeI fw	GGAATTCCATATGTTACCAAAAGCTACAGTAA	cloning of gene for MJ1647 into pET21a(+) (p1540)
**FW2341**	MJ1647 XhoI rv	GCGCGCTCGAGCTATAATTCAGATGTTATTACATTCAACA	cloning of gene for MJ1647 into pET21a(+) (p1540)
**FW1078**	MJ1647 dTM XhoI rv	GGGCTCGAGTCATCTCTTTAATCTTTCATCATCACACTG	cloning of gene for MJ1647ΔTM into pET21a(+) (p1542)
**FW693**	MJ1258 NdeI fw	GGAATTCCATATGGCTGAGCTTCCAGTTGC	cloning of gene for MJ1258 (A3) into pET21a(+) (p1539)
**FW2222**	MJ1258 XhoI rv	GCCGCTCGAGTTAACCTCTCAATGCTGCCTTT	cloning of gene for MJ1258 (A3) into pET21a(+) (p1539)
**FW2171**	MJ1647 K80E fw	AAATGCCAATATTAATTAAAGAGATGTTGA	Site-directed mutagenesis of p1540 to produce MJ1647 K80E
**FW2172**	MJ1647 K80E rv	CATCAGTTCTTTCACTAAGTTCCA	Site-directed mutagenesis of p1540 to produce MJ1647 K80E
**FW2178**	MJ1647 E95K fw	AATTATAGCTGACTCGAGC	Site-directed mutagenesis of p1540 to produce MJ1647 E95C (p1650)
**FW2179**	MJ1647 E95K rv	TAGATGTTATTACATTCAACA	Site-directed mutagenesis of p1540 to produce MJ1647 E95C (p1650)
**FW2327**	MJ1647 C28S/C62S vector fw	AGATATTAAACAGTCTGATGATGAAAGATTAAAGAGAAAGA	NEBuilder Hifi assembly on p1540 to produce MJ1647 C28S/C62S (p1737)
**FW2328**	MJ1647 C28S/C62S vector rv	CTTCAAGCATATTAGAGAGTTCATCAACCGCCTCT	NEBuilder Hifi assembly on p1540 to produce MJ1647 C28S/C62S (p1737)
**FW2329**	MJ1647 C28S/C62S insert fw	GGTTGATGAACTCTCTAATATGCTTGAAGAAATTATAAAG	NEBuilder Hifi assembly on p1540 to produce MJ1647 C28S/C62S (p1737)
**FW2330**	MJ1647 C28S/C62S insert rv	ATCTTTCATCATCAGACTGTTTAATATCTCTTGCTT	NEBuilder Hifi assembly on p1540 to produce MJ1647 C28S/C62S (p1737)
**FW2331**	MJ1647 K80C/E95C fw	TGTTGAATGTAATAACATCTTGTTTATAGCTGACTCGAGCACCA	Site-directed mutagenesis of p1737 to produce MJ1647 C28S/C62S/K80C/E95C (p1740)
**FW2332**	MJ1647 K80C/E95C rv	TCTCTTTAATTAATATTGGCATACAATCAGTTCTTTCACTAAGTTCCA	Site-directed mutagenesis of p1737 to produce MJ1647 C28S/C62S/K80C/E95C (p1740)
**FW2333**	MJ1647 E95 fw	TGTTGAATGTAATAACATCTGAATTATAGCTGACTCGAGCACCA	Site-directed mutagenesis with FW2332of p1737 to produce MJ1647 C28S/C62S/K80C (p1739)
**FW2334**	MJ1647 K80 rv	TCTCTTTAATTAATATTGGCATCTTATCAGTTCTTTCACTAAGTTCCA	Site-directed mutagenesis of p1737 with FW2331 to produce MJ1647 C28S/C62S/E95C (p1742)
**FW2240**	MJ1647 XhoI rv2	GCGCGCTCGAGTCATAATTCAGATGTTATTACATTCAACA	cloning of gene for MJ1647 with FW697 into pETDuet-1 (p1689)
**FW2192**	MJ1258 NcoIFor	GCGCGGCCATGGCTGAGCTTCCAGTTGC	cloning of gene for MJ1258 (A3) into pETDuet-1 (p1689)
**FW2243**	MJ A3 stp BamHI rv	GCCGGGATCCTTAACCTCTCAATGCTGCCTTT	cloning of gene for MJ1258 (A3) into pETDuet-1 (p1689)
Amplification and cloning of templates for EMSA, in vitro transcription and chromatin reconstitution assays			
**FW1137**	rpo5 operon fw	CATATGACTGTAAAAAGTATGGAGTTGATTTAATATTTT	Cloning of *rpo5* operon into pGEM for agarose EMSAs (p1664)
**FW1138**	rpo5 operon rv	CTCGAGTCTTTAGCCACAACCTCTGGA	Cloning of *rpo5* operon into pGEM for agarose EMSAs (p1664)
**FW1522**	T6 rpo5 NcoI fw	GCGGCCATGGAAGAATTACAATGGCTGCAG	Cloning of 500 bp part of *rpo5* operon into pGEM containing T6 promoter (p1471)
**FW1523**	T6 rpo5 BamHI rv	GCGGGGATCCGTTTAACTTGAATTGTTATC	Cloning of 500 bp part of rpo5 operon into pGEM containing T6 promoter (p1471)
	30 bp fw	CATACTGGTTCCAAAGCATGAAATAGTTCCAA	EMSAs
	30 bp rv	TTGGAACTATTTCATGCTTTGGAACCAGTATG	EMSAs
	60 bp fw	CATACTGGTTCCAAAGCATGAAATAGTTCCAAAAGAAGAAGTTGAGGAG TTTTGAAGAGA	EMSAs
	60 bp rv	TCTCTTCAAAACTCCTCAACTTCTTCTTTTGGAACTATTTCATGCTTTG GAACCAGTATG	EMSAs

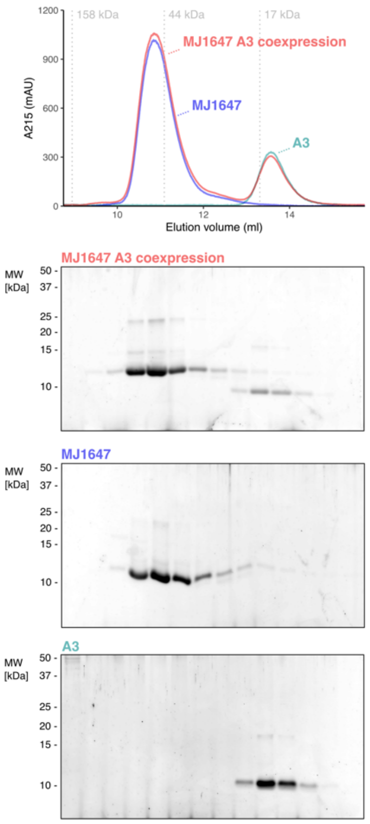

Supplementary Figure 1

SEC of recombinantly co-expressed MJ1647 and A3 versus the single proteins alone. Absorption profiles (top) and SYPRO-Orange-stained SDS-PAGE gels of the fractions (bottom) show distinct elution profiles for MJ1647 and A3 with no evidence of significant levels of cross-dimerisation between the proteins.

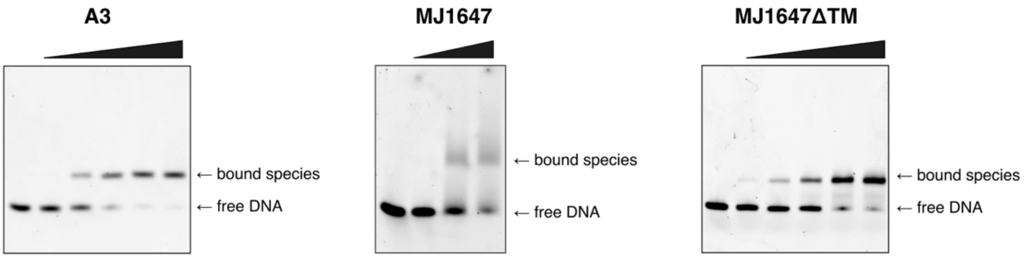

Supplementary Figure 2

EMSA experiments testing binding to 30 bp DNA templates. Increasing concentrations of histones A3, MJ1647 and the deletion variant MJ1647ΔTM were incubated with the respective ^{32}P-labelled DNA templates and resolved on native polyacrylamide gels. Protein concentrations were 2, 4, 6, 12, 14 μM histone monomers for A3 and MJ1647ΔTM, 4, 8, 12 μM for MJ1647.

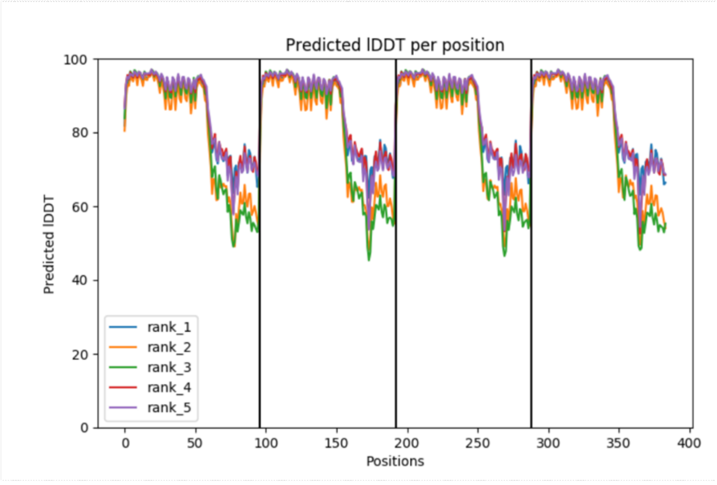

Supplementary Figure 3

Predicted local Distance Difference Test (plDDT) for the five MJ1647 tetramer models produced by AlphaFold2.

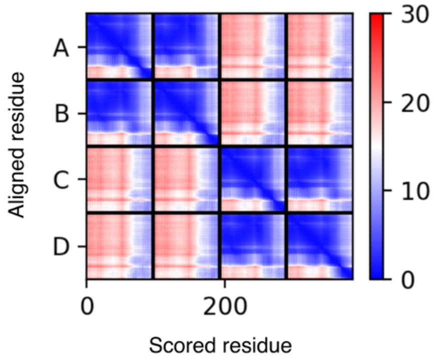

Supplementary Figure 4

Predicted alignment error (PAE) for the rank 1 MJ1647 tetramer model produced by AlphaFold2. A to D denote the four amino acid chains.

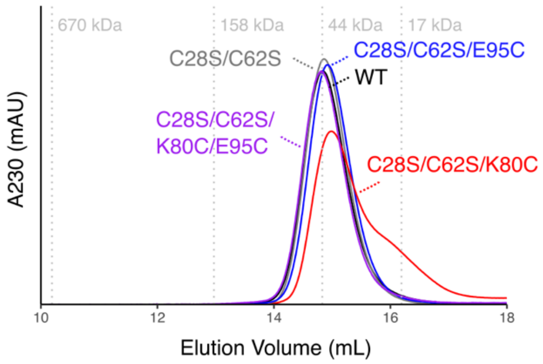

Supplementary Figure 5

Size exclusion chromatography elution profiles of MJ1647 cysteine mutants K80C, E95C, K80C/E95C in the MJ1647 C28S/C62S background compared to WT MJ1647.

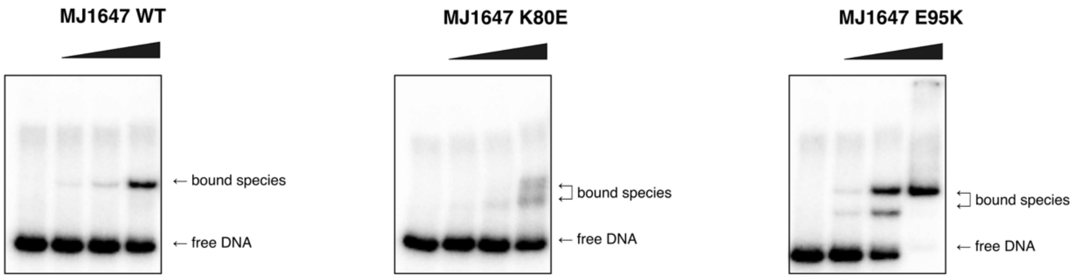

Supplementary Figure 6

MJ1647 K80E and E95K bind to 60 bp DNA as dimer at lower protein concentrations. EMSA experiments testing binding of 0.04 to 0.16 µM MJ1647 WT, K80E, and E95K monomers to 60bp ^{32}P-labelled DNA.

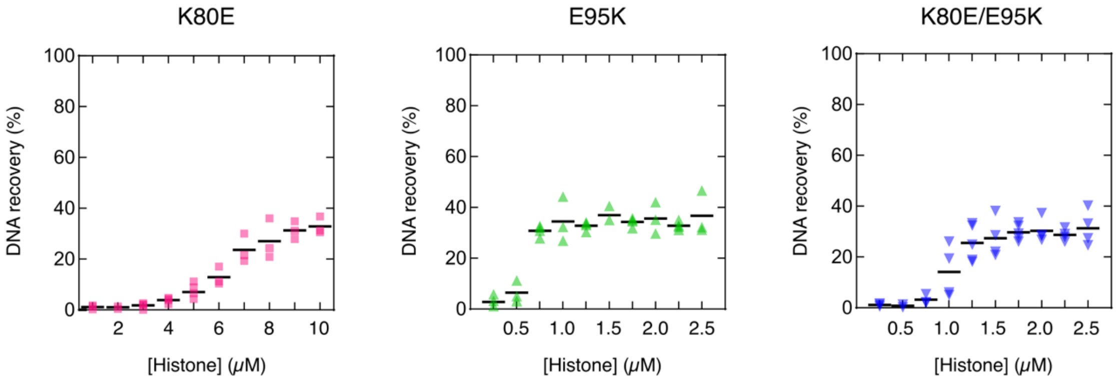

Supplementary Figure 7

MJ1647 K80E and E95K mutants retain DNA bridging activity. DNA bridging assays with salt bridge mutants of MJ1647 K80E and E95K and the double mutant K80E/E95K compared to wild-type MJ1647. DNA recovery (%) is plotted as a function of histone monomer concentration. Black bars show mean values of at least three independent measurements for each protein with colored symbols showing the individual replicates.

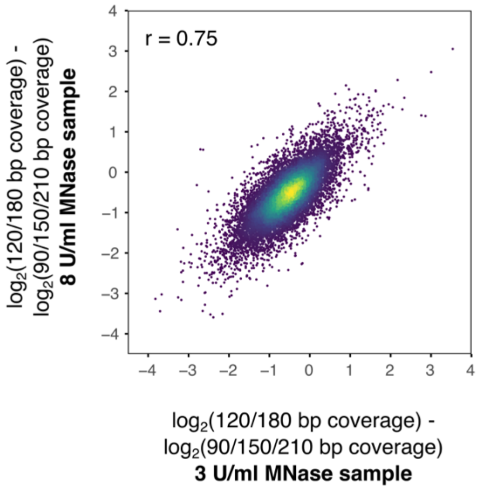

Supplementary Figure 8

MNase-seq data for *M. jannaschii* show coverage of both odd and even 30mer multiples for all genomic regions. The log_2-ratio of even 30mer multiple coverage (120 and 180 ± 5 bp DNA fragments) over odd 30mer multiple coverage (90, 150 and 210 ± 5 bp) was calculated for the 3u and 8u MNase-seq samples over consecutive 120 bp bins (n = 14810) covering the main chromosome and extrachromosomal elements. Bins with 0 coverage values were removed.

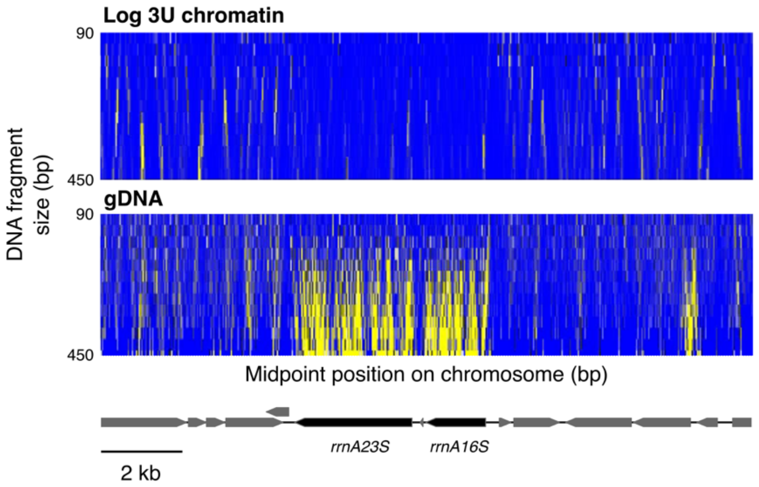

Supplementary Figure 9

rRNA loci are underchromatinised in M. jannaschii. MNase (3 units/ml) resistant mid-point frequency values from log-phase cells ranging in size from 90-450 bp in 30 bp intervals are plotted as a heatmap relative to a 16 kb region of the chromosome with positive frequency values coloured yellow. The rRNA operon genes are highlighted in black. The genomic DNA control MNase-seq data (gDNA) show increased signal across the rRNA operon likely due to the higher GC-content that counters the cutting preference of MNase.

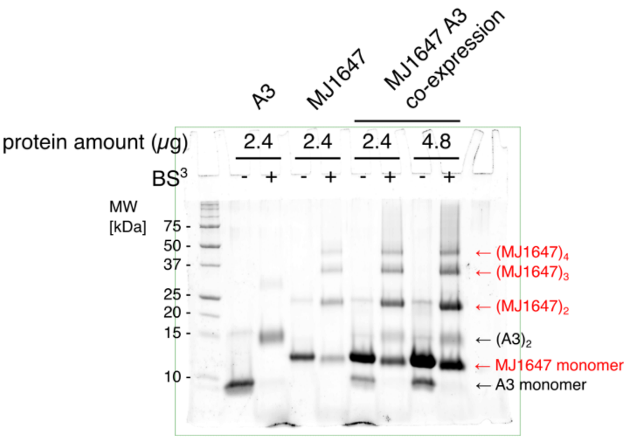

Supplementary Figure 10

Unedited/uncropped gel for Figure 1h

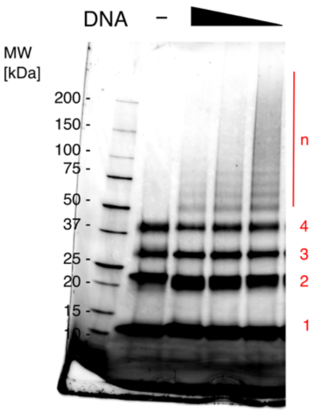

Supplementary Figure 11

Unedited/uncropped gel for Figure 1i

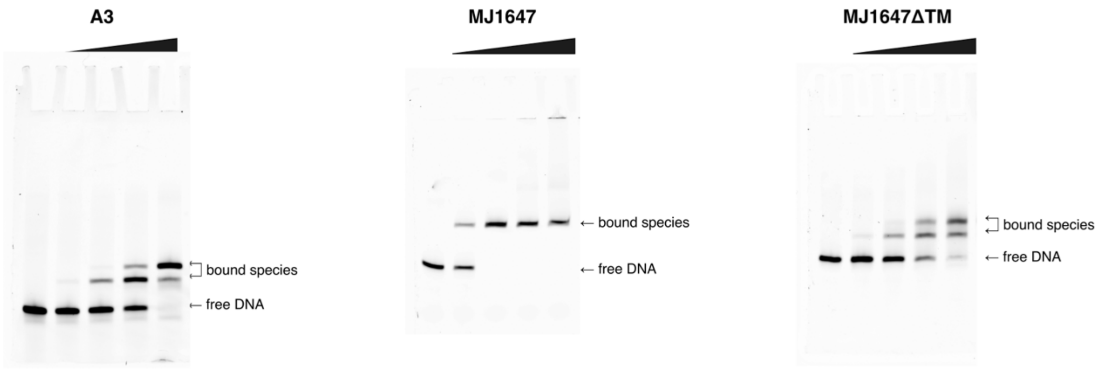

Supplementary Figure 12

Unedited/uncropped gels for Figure 2abc

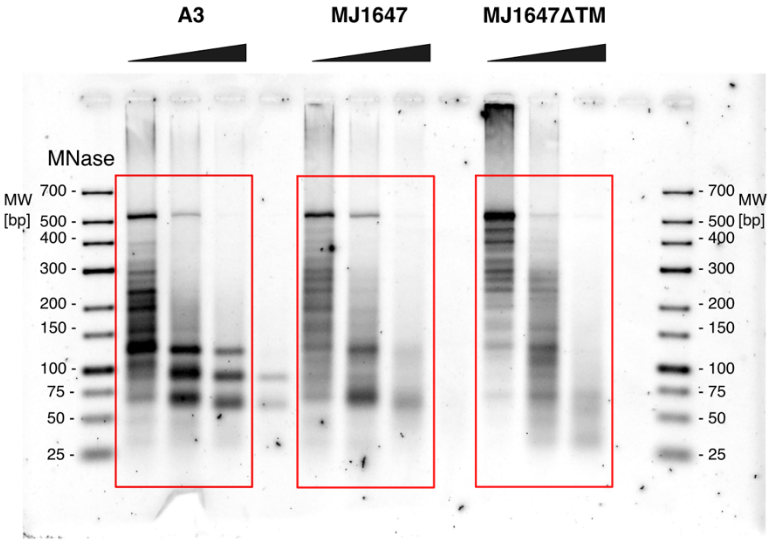

Supplementary Figure 13

Unedited/uncropped gels for Figure 2def

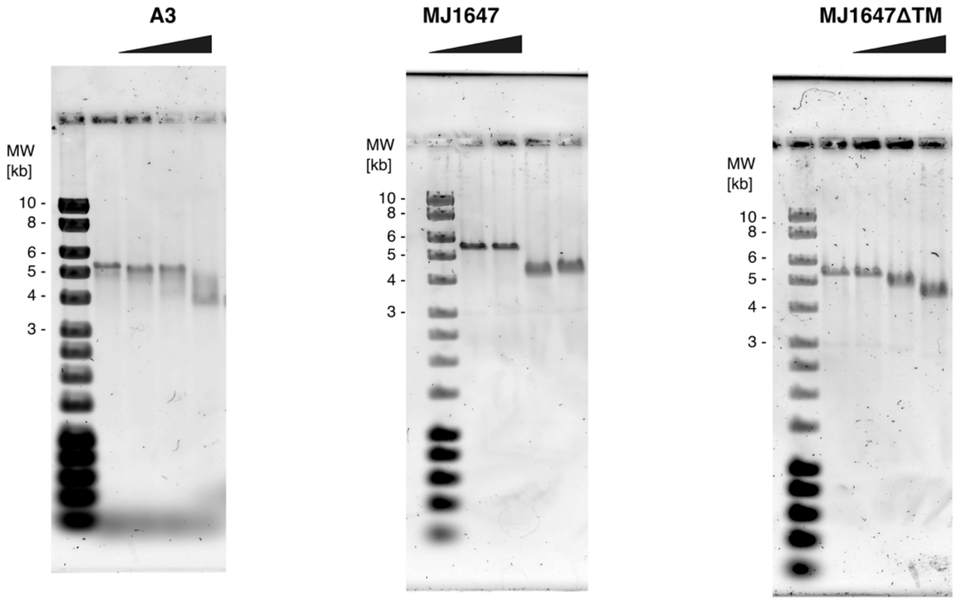

Supplementary Figure 14

Unedited/uncropped gels for Figure 2ghi

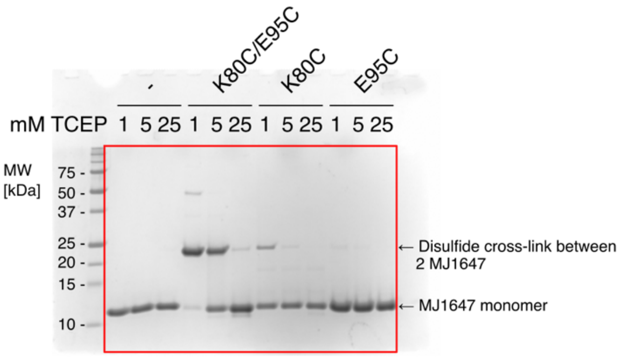

Supplementary Figure 15

Unedited/uncropped gel for Figure 4e

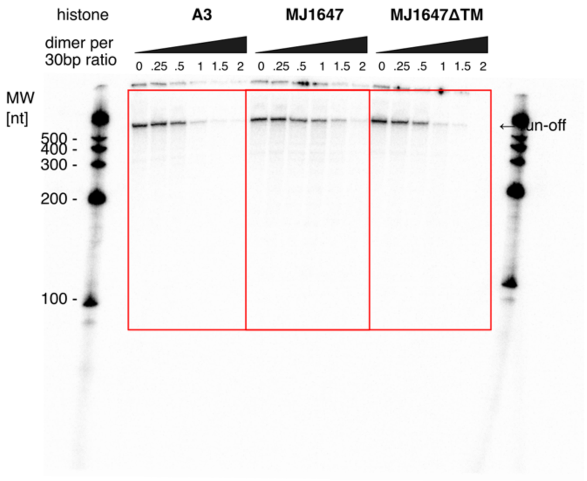

Supplementary Figure 16

Unedited/uncropped gel for Figure 6a

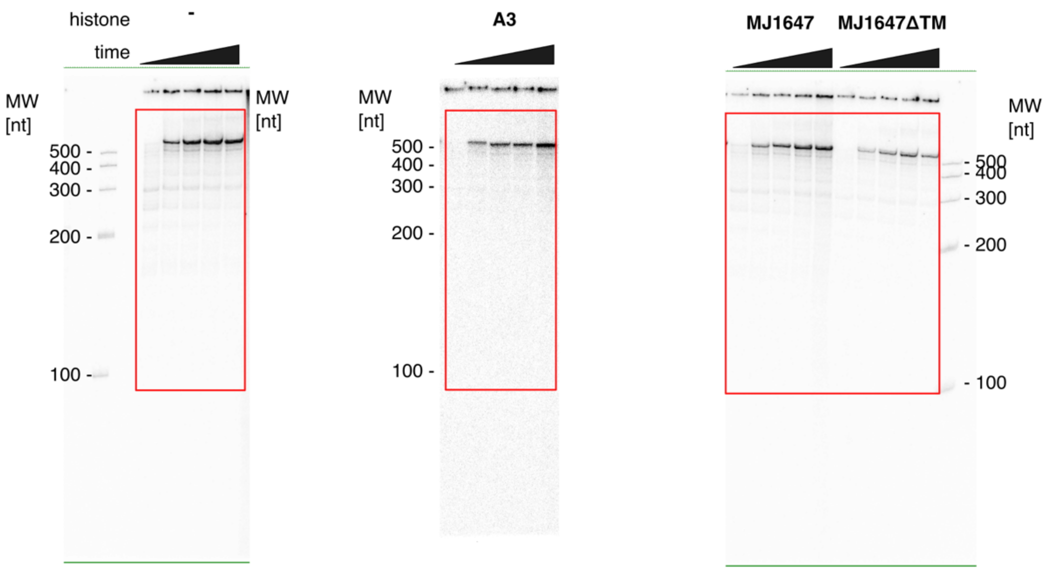

Supplementary Figure 17

Unedited/uncropped gel for Figure 6b

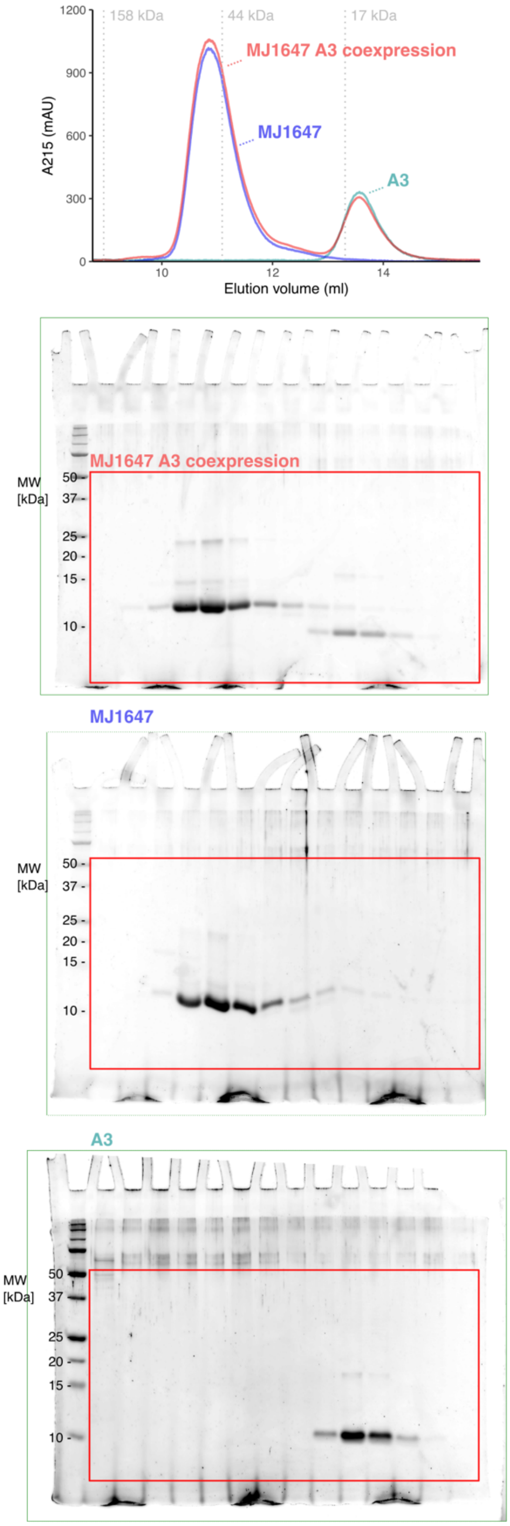

Supplementary Figure 18

Unedited/uncropped gel for Supplementary Figure 1

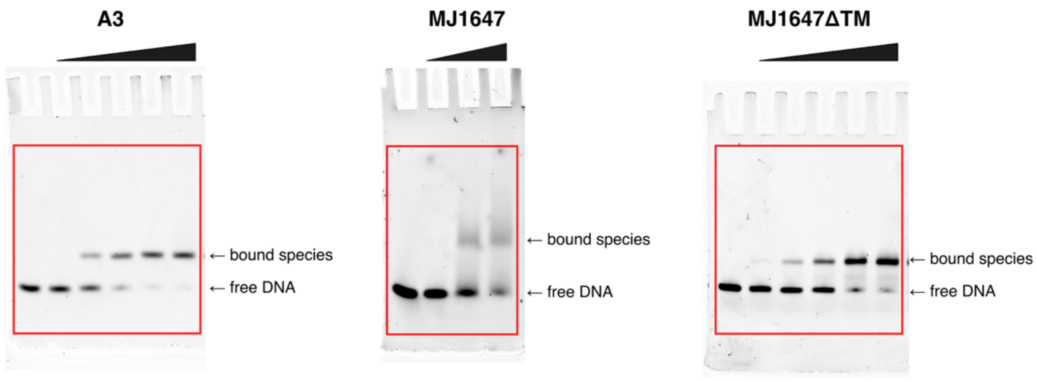

Supplementary Figure 19

Unedited/uncropped gel for Supplementary Figure 2

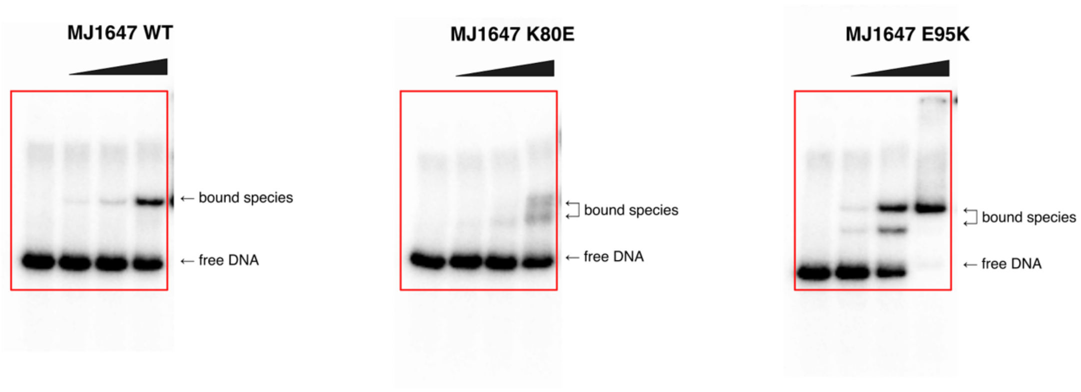

Supplementary Figure 20

Unedited/uncropped gel for Supplementary Figure 6
