# Supplementary material for: DNA-bridging by an archaeal histone variant via a unique tetramerisation interface
Source: Commun Biol. 2023 Sep 22;6:968. doi: 10.1038/s42003-023-05348-2 (PMC10516927; doi:10.1038/s42003-023-05348-2)
Supplement: Supplementary file 3 — Description of Additional Supplementary Files [file 42003_2023_5348_MOESM3_ESM.pdf]

Description of Additional Supplementary Files

File name: Supplementary Data 1
Description: The source data behind the graph in Figure 6a, multi-round transcription assays.

File name: Supplementary Data 2
Description: The source data behind the graph in Figure 6b, synchronized single-round transcription assays.
